# Stressful life events and coping style in Parkinson's disease patients before the initial diagnosis

**DOI:** 10.3389/fpsyg.2025.1435722

**Published:** 2025-06-25

**Authors:** Laura Sophie Neumaier, Ibrahim Raoua Ouedraogo, Wolfgang H. Jost

**Affiliations:** Parkinson Klinik Ortenau, Wolfach, Germany

**Keywords:** Parkinson's disease, etiology of PD, stress, coping, health prevention

## Introduction

To date there is only anecdotal evidence of the assumption that stressful life events (SLEs) and individual coping styles may have a direct effect not only on disease progression of PD but also on pathogenesis. In clinical practice patients often report about major life events having occurred several years or even immediately before the initial PD diagnosis. On closer examination it becomes apparent that they weren't able to cope well with this specific life events which in turn often led to the experience of immense stress. Patients report having the impression of this being the main cause for the development of their disease. Due to these reports it appears that a further examination of the relationship between SLEs, coping style and PD could contribute to gain a broader understanding of the pathogenic mechanisms that might lead to PD. This article will discuss the relationship between SLEs, coping style and health, and shed light on the role these variables might have in the etiology of PD. Finally, implications for clinical practice and health prevention are highlighted and recommendations for future research are given.

## Stressful life events, coping style and health

Stressful life events (SLEs) serve as predictors for onset, worsening and progression of several diseases (depression, asthma, autoimmune diseases etc.,) and have indirect effect on disease risk due to influence on physiology, as well as on affect and behavior (Cohen et al., 2019). Hypothalamic-pituitary-adrenocortical axis (HPA) and sympathetic-adrenal-medullary system (SAM), which are supposed to be activated in SLEs, increase the risk of physical and psychiatric disease if they happen frequently or a prolonged time (Cohen et al., 1995; McEwen, 1998). Based on the assumption that over lifespan most people will endure at least one Potentially Traumatic Event (PTE) (Kessler et al., 1985), similar to SLEs, the question arises why only a few of those experiencing such a critical event turn ill (see also Cohen et al., 2019). Coping might be one of the factors to play a crucial role. There is a wealth amount of evidence showing that coping style and health are closely related. According to Lazarus and Folkman (1984) coping means “the efforts to master, reduce, minimize or tolerate the negative consequences of internal or external demands.” They assumed coping to have a “moderator buffering effect,” meaning it diminishes the negative effects of stress on physic (Ogden, 1996; Wilkinson et al., 2000). Accumulating data suggest greater perceived control, greater self-efficacy, and lesser negative affectivity and rumination to be associated with psychological resilience in the face of stressful life events (reviewed in Adler and Matthews, 1994; Bonanno et al., 2011). Perceived self-efficacy or optimistic self-beliefs termed by Bandura (1995) refer to perceived competence to handle a stressful situation. Several studies have found that people with low feelings of mastery, self-esteem, or self-efficacy had a higher risk of mental (Tahmassian and Jalali Moghadam, 2011) and physical disorders (Zhou et al., 2021). The mechanism by which personal coping resources influence health is similar to those suggested for social support (McFadden et al., 2021). Personal coping resources (Bendezú et al., 2016) may directly influence various physiological responses and health-related behavior. In addition, in line with the stress-buffer model, personal coping resources were found to moderate the adverse effects of stressors on mental well-being (Li et al., 2022). Despite these observed associations, research into the effects of personal coping resources on health is limited. In contrast the link between social support and health has been of great interest and has been investigated in various studies (Chung et al., 2021). For PD, there are consistent indications of a better wellbeing among people with a large social network (Ghorbani Saeedian et al., 2014).

## Stress, coping and PD

In the literature we find increasing evidence that stress contributes to nigrostriatal degeneration (Djamshidian and Lees, 2014) in individuals who do not have a command over adequate coping mechanisms. Chronic stress or major stressful events lead to a hyperactivation of the HPA axis (McEwen, 2007) and play an essential role in disease development (DeMorrow, 2018). This association is well established in Alzheimer's for example (Rothman and Mattson, 2009). Stress leads to a greater risk of disease development as well as enhanced disease progression (Rothman and Mattson, 2009). As can be seen in the literature inflammatory processes play a large role in PD as well as in Alzheimer's (Hartmann et al., 2003; van Gool et al., 2003). In Djamshidian and Lees (2014) it is discussed that experience of unusual stress could be presymptomatic of neurodegeneration which further leads to structural and functional changes in some brain regions such as prefrontal, limbic and parietal areas and might find expression on a behavioral level, meaning difficulties in coping with SLEs. We suggest, in contrast, that the process occurs in reverse: it begins with maladaptive coping mechanisms, which lead to specific brain changes when the individual is confronted with stress. These changes, in turn, further reinforce the coping style employed. The coping style may influence whether a life event is perceived as stressful in the first place. Due to the limited evidence available, no definitive conclusions can be drawn. When applying the model proposed by Cohen (2004) to describe the pathways following a stressful life event, this aspect should be taken into consideration.

Djamshidian and Lees (2014) report an interesting case of two PD patients who showed full remission of PD-symptoms after the elimination of the chronic stressor. Several factors, toxic, infectious and iatrogenic, could be excluded as cause of their symptoms. The authors point out that the patients might have suffered from functional parkinsonism, which is sometimes not easy to distinguish from idiopathic PD. However, their good response to L-Dopa does not conform to the prototypical response of a diagnosed Parkinsonism patient (LaFaver and Espay, 2017). Even if one cannot be sure whether if stress caused their symptoms and the elimination of the stressor led to full remission, this observation could inspire further research investigating the association between stress and the development of PD symptoms.

Several authors (Hald and Lotharius, 2005; Djamshidian and Lees, 2014; Dias et al., 2013; Hou et al., 2014; Smith et al., 2008) have investigated the link between stress and PD, in particular in rats (e.g., Rasheed et al., 2009; Smith et al., 2008). Sugama et al. (2016) report on dopaminergic degeneration in rats exposed to chronic stress through elevated oxidative stress, induced by dopamine (Hald and Lotharius, 2005; Lotharius and Brundin, 2002a,b), as well as microglial activation which further led to inflammation, all processes assumingly contributing to PD pathogenesis (Dias et al., 2013; Smith et al., 2008). Stress not only contributes to disease development but also leads to an aggravation of PD symptoms such as tremors (Moore et al., 2001).

Furthermore, symptoms that include fatigue following a maladaptation to stress have a significant correlation to the PD development risk (Djamshidian and Lees, 2014). Likewise, symptoms that fall in the chronic fatigue spectrum resemble the non-motor symptoms in PD (Djamshidian and Lees, 2014). Considering the current evidence as well as anecdotal reports, it is imagined that the non-motor symptoms thought to precede PD by several years, if untreated, lead to PD. Research needs to be done to investigate a possible association.

Holmes and Rahe (1967) pointed out that not only negative life events, but also positive ones can be perceived as stressful and might require adaptation. This fits anecdotal reports of patients having experienced eustress over a longer time period, which then turned into distress with its negative effects on health.

## Coping style and brain changes

The prefrontal cortex, as well as amygdala and hippocampus are involved in stress response modulation (Radley et al., 2015) with inactivation of the medial prefrontal cortex being associated with an impaired stress response (Weinberg et al., 2010). A study with tinnitus patients showed that changes in the dorsolateral prefrontal cortex are associated with maladaptive coping and an increased activity in the Default Mode Network (DMN) could be observed in patients using maladaptive coping (Vanneste et al., 2014). Furuyashiki and Kitaoka (2019) wrote that prefrontal dopamine pathways and inflammation in the brain and body lead to adaptive or maladaptive coping. We suggest in contrast that the coping style decides on the activation of the specific pathways. To sum up, stronger PFC activation and lower DMN activation are associated with successful emotion regulation (Steinfurth and Hamm, 2022). Mindfulness exercises such as meditation lead to a thickening of prefrontal areas and reducing amygdala as well as DMN activation, and this could be an efficient tool leading to healthy coping.

The evidence in terms of the efficacy of a specific coping style is inconclusive. There are several studies showing that emotion-focused coping is positively correlated to distress (Sanders-Dewey et al., 2001; Moore and Seeney, 2007) whereas in others it is labeled as adaptive coping strategy (Machado et al., 2020). Passive coping leads to more health dysfunction in Parkinson's disease patients (Schreurs et al., 2000). Both emotion-focused and problem-focused coping are associated with higher optimism (Anzaldi and Shifren, 2018) and are therefore useful when applied appropriately and context-dependent (Folkman and Lazarus, 1980). As adaptive coping leads to brain changes also seen after meditation exercises one can assume that a “mindful” coping style would be ideal involving acknowledgment of all emotions without judgment, as well as accepting what cannot be changed and changing where change is possible.

## Implications for research and clinical practice

Evidence indicates that stress and coping play an important role in pathogenesis and disease progression of PD. Further research on the exact mechanisms by which coping and stressful life events contribute to the pathogenesis of PD, as well as research on the implementation of prevention strategies are needed. Interventions should be implemented at an early stage. These include psychoeducation about healthy coping styles and adequate stress management. A mindful healthy lifestyle including sports—as exercise has been shown to have a neuroprotective effect—as well as mindfulness exercises are of great importance. There is evidence that meditation promotes adequate coping with stress through enhancing openness to experience (Pokorski and Suchorzynska, 2017). It would be worthwhile investigating the effect of mindfulness interventions, such as meditation, on coping and disease progression in PD patients. Meditation has been shown to lead to functional and structural changes in the brain which are beneficial for emotion regulation (Esch, 2013) as well as the maintenance of balance in neurotransmitters which is essential for cognition and physiology (Krishnakumar et al., 2015). Moreover, there is evidence that neurotransmitters such as dopamine increase by meditating (Esch, 2013; Kjaer et al., 2002). Not only in preventive medicine but also in treating the non-motor symptoms of Parkinson's, among other neuropsychiatric disorders such as depression, anxiety and cognitive impairment, mindfulness interventions (meditation, ACT—Acceptance and Commitment Therapy, MBCT—Mindfulness-Based Cognitive Therapy, and DBT—Dialectical Behavior Therapy) could be beneficial and deserve further investigation.
